# Supplementary material for: Deletion of wfs1 Impairs Oligodendrocyte Precursor Cells Dorsal Distribution and Myelination Through the wfs1-hmgcs1 Axis in Zebrafish
Source: Biology (Basel). 2026 Aug 16;15(16):1402. doi: 10.3390/biology15161402 (PMC13510151; doi:10.3390/biology15161402)
Supplement: Supplementary file 1 [file biology-15-01402-s001.zip › File S1-Transcriptome sequencing data and differentially expressed genes (DEGs) in wfs1-- zebrafish at 5 dpf/1.GO/wfs1KOvsWT/GSEA/wfs1KO/SNRNA_3__END_PROCESSING/SNRNA_3'_END_PROCESSING(GO_0034472).html]

Details for gene set SNRNA\_3'\_END\_PROCESSING(GO:0034472)[GSEA]


|  || Dataset | deg.sample |
| Phenotype | sample.cls |
| Upregulated in class | wfs1KO |
| GeneSet | SNRNA\_3'\_END\_PROCESSING(GO:0034472) |
| Enrichment Score (ES) | 0.57042193 |
| Normalized Enrichment Score (NES) | 1.8717366 |
| Nominal p-value | 0.0 |
| FDR q-value | 0.091542326 |
| FWER p-Value | 0.444 |
Table: GSEA Results Summary

  

Fig 1: Enrichment plot: SNRNA\_3'\_END\_PROCESSING(GO:0034472)
  
Profile of the Running ES Score & Positions of GeneSet Members on the Rank Ordered List

  

| PROBE | DESCRIPTION   (from dataset) | GENE SYMBOL | GENE\_TITLE | RANK IN GENE LIST | RANK METRIC SCORE | RUNNING ES | CORE ENRICHMENT || 1 | 565000 | exosc3 |  |  | 470 | 3.081 | 0.3627 | Yes |
| 2 | 751632 | exosc5 |  |  | 1460 | 1.686 | 0.5382 | Yes |
| 3 | 327593 | vwa9 |  |  | 3814 | 0.866 | 0.5704 | Yes |
| 4 | 550247 | exosc7 |  |  | 10822 | 0.254 | 0.3817 | No |
| 5 | 393406 | ddx26b |  |  | 11091 | 0.239 | 0.4026 | No |
| 6 | 393712 | exosc4 |  |  | 14263 | 0.080 | 0.3129 | No |
| 7 | 286776 | ints7 |  |  | 15053 | 0.043 | 0.2934 | No |
| 8 | 553794 | ints8 |  |  | 15410 | 0.027 | 0.2856 | No |
| 9 | 562131 | ints1 |  |  | 15864 | 0.004 | 0.2718 | No |
| 10 | 568260 | toe1 |  |  | 16214 | -0.008 | 0.2619 | No |
| 11 | 564642 | ints5 |  |  | 17880 | -0.071 | 0.2183 | No |
| 12 | 569456 | ints6 |  |  | 18541 | -0.101 | 0.2099 | No |
| 13 | 450057 | exosc9 |  |  | 18931 | -0.119 | 0.2123 | No |
| 14 | 323016 | exosc8 |  |  | 19222 | -0.132 | 0.2193 | No |
| 15 | 445066 | usb1 |  |  | 20710 | -0.199 | 0.1970 | No |
| 16 | 550234 | exosc2 |  |  | 23270 | -0.346 | 0.1591 | No |
| 17 | 553563 | ints2 |  |  | 23986 | -0.391 | 0.1845 | No |
| 18 | 415251 | ints12 |  |  | 25876 | -0.518 | 0.1887 | No |
Table: GSEA details
[plain text format]

  

Fig 2: SNRNA\_3'\_END\_PROCESSING(GO:0034472)
  
Blue-Pink O' Gram in the Space of the Analyzed GeneSet

  

Fig 3: SNRNA\_3'\_END\_PROCESSING(GO:0034472): Random ES distribution
  
Gene set null distribution of ES for
 **SNRNA\_3'\_END\_PROCESSING(GO:0034472)**
